# Supplementary material for: Intra- and inter-rater reliability, agreement, and minimal detectable change of the handheld dynamometer in individuals with symptomatic hip osteoarthritis
Source: PLoS One. 2023 Jun 8;18(6):e0278086. doi: 10.1371/journal.pone.0278086 (PMC10249871; doi:10.1371/journal.pone.0278086)
Supplement: Attachment — Submitted filename: Response to Reviewers.docx [file pone.0278086.s001.docx]

___________________________________________

Emily Chenette

Editors in Chief

PLOS ONE

March 12, 2023

Dear Editor,

Revision of the manuscript: PONE-D-22-30852

Title: "Intra- and inter-rater reliability, agreement, and minimal detectable change of the handheld dynamometer in individuals with symptomatic hip osteoarthritis".

Thank you for the opportunity to resubmit our manuscript. The comments from the reviewers were highly insightful and enabled us to improve the quality of our manuscript. We have made relevant changes according to their advice. A point-by-point response to the reviewers, as well as the marked-up version (highlighted in yellow) of the manuscript, are attached.

Thank you again for your time and effort in considering this manuscript for publication.

Yours sincerely,

**EDITOR TEAM COMMENTS BELOW:**

**Reviewer #1:** It is a very interesting study. I think that it is highly significant to appropriately evaluate the hip joint muscle strength of people with hip osteoarthritis. Please correct according to comments from the editorial committee.

**L391-**　As a result of the hip flexion, examiner B, who weighs 68 kg, may not be able to fix HHD at less than 25% of his body-weight. It is possible that this tendency may become more pronounced in examiners weighing 68 kg or less. I think there are a lot of cases where the weight of the examiner is 68 kg or less. Therefore, the reliability of hip flexion is questionable. If the examiner's body weight is even smaller, it may affect the results of other measurement items. Therefore, the weight of the examiner to whom the results of this study are applicable is considered to be limited.

**Answer:** Dear reviewer, thank you very much for this important comment. The use of a handheld dynamometer is very simple, and the rater's ability to resist hip strength is a very relevant point to be highlighted, since lighter raters or even evaluations in conditions such as with healthy individuals may interfere with the reproducibility of measures. However, we partially agree with your argument, as we believe that a trained assessor, with knowledge of biomechanics and positioning, may be able to perform measurements with adequate consistency, with a minor influence of his/her body weight. To clarify this limitation, we have added in line 415 a suggestion for caution concerning extrapolation of our results to raters lighter than 68kg, presuming difficulties in resisting during the tests and consequently lower agreement between measures and raters, and suggesting intensive training to achieve better intrarater reliability.

**L378 –** The random error between examiners is around 5%, but considering that the random error within examiners is 5-20%, how should we measure in clinical practice? This is an important interpretation that will lead to clinical practice, so please add it.

**Answer:** Thank you for your helpful comment. We have tried to address the issue above in line 389, emphasizing the improvement in MDC% when considering two measures in the inter-rater analysis, suggesting that the mean of at least two measurements should be used to achieve appropriate agreement and consistency.

**L207-**　Are the ICCs used in this study ICC(1,1) and (2,1)?

**Answer:** We appreciate the question raised, as this important information was not clear in the first version of the manuscript. We used ICC (2,1) in our statistical analysis. To make this information available in the methods, we have added information in the Statistical Analysis (line 213) and table 2 legend (line 278 and 282).

**L217**-　In this study, the significance level is set to 5%, so I think it is better to use MDC95 as the MDC. The random error increases accordingly, but it is also balanced with the 95% CI of the ICC.

**Answer:** Thank you for your suggestion. We decided to revise the report according to your request. We had some doubts about how rigorous we should be when calculating the MDC% for manual dynamometry and its impact on clinical practice. Indeed, as expressed in table 2 (page 13), the change in CI elevated MDC and MDC% for both raters in the intra-rater analysis, mainly for rater B. However, the inter-rater comparison was still considered very satisfactory and applicable in the clinical setting. Thus, changes have been made in line 42 to point to the two variables that were not under the 10% limit; in lines 220 and 222 regarding the MDC equation; table 2 was adequate to show values with 95%CI (title, legend, and content on 7^th^, 12^th^, and 17^th^ column); in line 305, without changes in the conclusion, and in line 389, in agreement with the alterations proposed in your second suggestion.

**L299-**　Although we consider systematic errors by the Bland-Altman method, please use the terms fixed error for the LOA and proportional error for the regression equation.

**Answer:** Dear reviewer, in an effort to use the correct terms considering the Bland-Altman method, we have made some changes in lines 316, 322, 406, 407, and 409.

___________________________________________________________________________________

**Reviewer #2**: Congratulate the authors for their proposal and article. Some minor comments:

**1)** The graphics don't look good.

**Answer:** Dear reviewer, thank you for your attention regarding the graphics. We have reformulated them to give readers clearer and more precise information, including the 95%CI for bias and LoA, according to the formula provided by Giavarina et al (2015). In addition, we have improved the quality of the image. The figure is resubmitted with alterations.

Reference: Giavarina D. Understanding Bland Altman analysis. Biochem Med (Zagreb) 2015; 25:141–51. https://doi.org/10.11613/BM.2015.015.

**2)** I recommend being consistent with the use of the point for decimals (bug in ln 292).

**Answer:** Thank you very much for the comment. We have carefully reviewed the manuscript, and changes have been made in lines 301 and 302, to correct the use of the point for decimal numbers.

**3)** I recommend providing images of the tests for example (<https://doi.org/10.1080/1091367X.2020.1822363>):

**Answer:** Thank you again for your recommendation. We appreciate your thoughtful suggestion. To illustrate the protocol sequence we used, we have added a figure composed of four parts, to allow readers to visualize the position of the rater and participant during the test routine, named Fig1 (A-D). As a consequence, the corresponding legend has been added in line 181, references to this figure have been added in lines 161, 167, 171, and 175, and the title and legend of the graphics have been renamed as figure 2.
