# Supplementary material for: Intra- and inter-rater reliability, agreement, and minimal detectable change of the handheld dynamometer in individuals with symptomatic hip osteoarthritis
Source: PLoS One. 2023 Jun 8;18(6):e0278086. doi: 10.1371/journal.pone.0278086 (PMC10249871; doi:10.1371/journal.pone.0278086)
Supplement: Attachment — Submitted filename: Response to reviwers.docx [file pone.0278086.s002.docx]

___________________________________________

Emily Chenette

Editor in Chief

PLOS ONE

May 7, 2023

Dear Editor,

Revision of the manuscript: PONE-D-22-30852R1

Title: "Intra- and inter-rater reliability, agreement, and minimal detectable change of the handheld dynamometer in individuals with symptomatic hip osteoarthritis".

We would like to thank you for the opportunity to resubmit our manuscript. The comments from the reviewers and references sent were very helpful and enabled us to further improve the quality of our manuscript. We have made some changes according to your advice. A point-by-point response to the reviewers, as well as the marked-up version (highlighted in yellow) of the manuscript, are attached.

We appreciate the reviewer’s time and attention in considering this manuscript for publication.

Yours sincerely,

**EDITOR TEAM COMMENTS BELOW:**

**Reviewer’s comment #1:** The reply to Reviewer 1's point regarding raters of <68kg mass, is more informative than what was included in the manuscript. Please expand in the manuscript on this limitation in line with your response, as it is a crucial point.

**Answer:** Dear reviewer, thank you for pointing out that the information in the manuscript was not sufficiently clear in our previous response letter. We have now added a new statement regarding the limitations of this paper in lines 421-427 aligned with our previous response, as requested.

We have expanded our previous statement from: “Considering that rater B, who weighs 68kg, had some difficulty stabilizing the HHD for hip flexor measurements, we suggest that lighter raters should be intensively trained to achieve better intra-rater reliability” to the following phrase: “The rater's ability to resist hip strength is a very relevant point that could interfere with the reproducibility of measurements [1,2]. Considering that rater B, who weighs 68kg, had some difficulty stabilizing the HHD for hip flexor measurements, we suggest that lighter raters should be intensively trained to achieve better consistency and to rigorously follow the standardized protocol, since it is possible that knowledge of biomechanics and positioning may overcome the influence of his/her body weight and presumed strength [3]”.

References:

1- Kelln BM, McKeon PO, Gontkof LM, Hertel J. Hand-held dynamometry: reliability of lower extremity muscle testing in healthy, physically active,young adults. J Sport Rehabil 2008;17:160–70. https://doi.org/10.1123/jsr.17.2.160.
2- Krause DA, Neuger MD, Lambert KA, Johnson AE, DeVinny HA, Hollman JH. Effects of examiner strength on reliability of hip-strength testing using a handheld dynamometer. J Sport Rehabil 2014;23:56–64. <https://doi.org/10.1123/jsr.2012-0070>.
3- Morin M, Hébert LJ, Perron M, Petitclerc É, Lake SR, Duchesne E. Psychometric properties of a standardized protocol of muscle strength assessment by hand-held dynamometry in healthy adults: a reliability study. BMC Musculoskelet Disord. 2023 Apr 14;24(1):294. doi: 10.1186/s12891-023-06400-2. PMID: 37060020; PMCID: PMC10103411.

**Reviewer’s comment #2:** You discuss the reduction in error by averaging the two trials and state that this makes the measurement sufficiently sensitive to detect clinically meaningful changes, but provide no reference/reminder of what those changes are (and thus that the averaged measurements error allows detection of such changes). Reference 3 might be sufficient - but you need to provide a 'yardstick' for that statement.

**Answer:**

Dear reviewer, we have expanded our discussion by adding new references to anchor our discussion concerning the number or measurements, as stated in lines 397-401. Our results suggest that the protocol of measurement with HHD is reliable. We consider the mean of two measures, as the results expressed higher values of ICC when comparing raters. Sources of variability may result from the measuring instrument, raters, or characteristics of the measure taken [1,2]. Biological variations in generating peak force, such as muscle fibers, elicited fatigue, limb and device positioning, stabilization of the HHD, attention of subject/raters, environmental conditions and symptoms related to the disease, and others, may influence the measurements taken. When averaging results across trials, the sources of variability between these measurements are theoretically reduced, as stated by Portney and Watkins (2015). The authors described that random error is not related to the true score when enough measurements are taken. Positive random errors would eventually cancel negative ones, making the average scores a reasonable estimate of the true score. Therefore, averaging two measures would help to reduce variability and consequently improve ICC values [1]. This is consistent with a practical protocol of measurements that could easily be reproduced in a clinical scenario. It can also minimize the time requirements and reduce discomfort/pain from repeated strength tests in a compromised joint, such as a hip with osteoarthritis.

Regarding what was described in the discussion section: “seems to be reliable for clinical purposes since it can detect small variations that could be attributed to a real clinical change”; We have rephrased this statement, excluding the word “clinical”, to make sure that readers do not mistake it with the minimal clinical important difference (MCID) [3,4], since we refer to the minimal detectable change (MDC) that could be attributed to a real difference of peak force and average peak force instead of a difference resulting from random error.

References:

1- Portney, Leslie Gross MPW. Foundations of Clinical Research: Applications to Practice. 3rd ed. Upper Saddle River, New Jersey: Pearson/Prentice Hall; 2015.
2- Bialocerkowski, A. E., & Bragge, P. (2008). Measurement error and reliability testing: Application to rehabilitation. *International Journal of Therapy and Rehabilitation*, *15*(10), 422–427. <https://doi.org/10.12968/ijtr.2008.15.10.31210>
3- Suijker, J. J., Van Rijn, M., Ter Riet, G., van Charante, E. M., De Rooij, S. E., & Buurman, B. M. (2017). Minimal important change and minimal detectable change in activities of daily living in community-living older people. The journal of nutrition, health & aging, 21, 165-172.
4- Turner, D., Schünemann, H. J., Griffith, L. E., Beaton, D. E., Griffiths, A. M., Critch, J. N., & Guyatt, G. H. (2010). The minimal detectable change cannot reliably replace the minimal important difference. Journal of clinical epidemiology, 63(1), 28-36.

**Reviewer’s comment #3:** The statement in Lines 273-275 are the increased pain not affecting the measurement can only hold true if you knew that the participants truly performed at maximum capacity; however, this is not possible to know. For example, it could be that the increased pain in the second trial resulted in lower force generation (e.g. <https://ddec1-0-en-ctp.trendmicro.com:443/wis/clicktime/v1/query?url=https%3a%2f%2fwww.frontiersin.org%2farticles%2f10.3389%2ffpsyg.2010.00210%2ffull&umid=d55a5243-5760-49d2-acea-601c4247b06a&auth=6b639a990a359ff1d6cc8761081d57748ce3c81e-650e099fe74b65ee9f9331a4049e4dd2d0c53860>) and, therefore, a closer score to the first trial which would, in turn, result in higher ICC but through a submaximal second trial and a maximal first trial. On the other hand, it could be that the difference of <1 cm in pain is not clinically significant / meaningful (e.g. [https://ddec1-0-en-ctp.trendmicro.com:443/wis/clicktime/v1/query?url=https%3a%2f%2fpubmed.ncbi.nlm.nih.gov%2f9428860%2f&umid=d55a5243-5760-49d2-acea-601c4247b06a&auth=6b639a990a359ff1d6cc8761081d57748ce3c81e-80734d227bdb7133929fdb958254a41191226b88](https://ddec1-0-en-ctp.trendmicro.com/wis/clicktime/v1/query?url=https%3a%2f%2fpubmed.ncbi.nlm.nih.gov%2f9428860%2f&umid=d55a5243-5760-49d2-acea-601c4247b06a&auth=6b639a990a359ff1d6cc8761081d57748ce3c81e-80734d227bdb7133929fdb958254a41191226b88)). Please revise that point to remove the statement you currently have.

**Answer:** We would like firstly to thank you for the references suggested. They were well-conducted studies and essential to discuss our data. We have performed a modification following your comment. We searched the literature to verify if, beyond the imprecision of VAS for pain around ± 20mm as pointed out by DeLoach et al. (1998), there was some definition of MCID concerning our study population. We found that a clinical perceived difference in individuals with hip osteoarthritis depends on the baseline value. Therefore, if we consider the first trial as a baseline, values between the interval of 50-65 would require a difference of 24mm to be considered clinically important, and for baseline values > 65mm, a clinically perceived difference must be even higher, 30mm. Thus, the statistical difference between the two trials of rater A was not high enough to be considered clinically relevant. Comparing the first and second trials of rater B, differences were neither statistically significant nor clinically meaningful, similar to the interrater comparison. In conclusion, besides differences that were not clinically relevant, the test protocol was still painful, and statements regarding the influence of pain on the capability of generating maximal force in all trials and, consequently the instrument reliability could not be inferred in this study design. We have removed the statement: “Although there was an existing difference in VAS for pain intensity after the rater A test compared to the retest, it did not seem to have a relevant effect on intra-rater ICC, since rater A presented better ICC and lower SEM values than rater B”; and added some comments in line 273-277 according to your suggestion.

References:

1- Stauffer ME, Taylor SD, Watson DJ, Peloso PM, Morrison A. Definition of Nonresponse to Analgesic Treatment of Arthritic Pain: An Analytical Literature Review of the Smallest Detectable Difference, the Minimal Detectable Change, and the Minimal Clinically Important Difference on the Pain Visual Analog Scale. Int J Inflam 2011; 2011:1–6. <https://doi.org/10.4061/2011/231926>.
2- Tubach F, Ravaud P, Baron G, Falissard B, Logeart I, Bellamy N, et al. Evaluation of clinically relevant changes in patient reported outcomes in knee and hip osteoarthritis: the minimal clinically important improvement. Ann Rheum Dis 2005; 64:29–33. <https://doi.org/10.1136/ard.2004.022905>.
